# Microbiome–transcriptome analysis reveals that dietary supplementation with macleaya cordata extract alters multiple immune pathways with minimal impact on microbial structure

**DOI:** 10.3389/fcimb.2023.1264550

**Published:** 2023-09-29

**Authors:** Jian Wu, Xiaoli Zhang, Chuanshe Zhou, Jinzhen Jiao, Zhiliang Tan

**Affiliations:** ^1^ CAS Key Laboratory of Agroecological Processes in Subtropical Region, National Engineering Laboratory for Pollution Control and Waste Utilization in Livestock and Poultry Production, Hunan Provincial Key Laboratory of Animal Nutritional Physiology and Metabolic Process, Institute of Subtropical Agriculture, The Chinese Academy of Sciences, Changsha, Hunan, China; ^2^ College of Advanced Agricultural, University of Chinese Academy of Sciences, Beijing, China

**Keywords:** host-microbe interactions, antibiotics, *Macleaya cordata* extract, gene regulation, microbiome

## Abstract

**Background:**

As a potential antibiotic alternative, macleaya cordata extract (MCE) has anti-inflammatory, antioxidant, and antimicrobial properties. This study was conducted to assess the impact of MCE supplementation on the gut microbiota and its interplay with the host in young goats. Thirty female black goats with similar body weight (5.63 ± 0.30 kg) were selected and randomly allotted into one of three diets: a control diet (Control), a control diet with antibiotics (Antibiotics, 21 mg/kg/day vancomycin and 42 mg/kg/day neomycin), and a control diet with MCE (MCE, 3.75% w/w premix).

**Results:**

Principal coordinate analysis of the microbial community showed that samples of Antibiotic clustered separately from both Control and MCE (*p* < 0.001). The random forest analysis revealed that, in comparison to the Control group, the impact of Antibiotics on the microbiota structure was more pronounced than that of MCE (number of featured microbiota, 13 in Antibiotics and >6 in MCE). In addition, the pathways of significant enrichment either from DEGs between Antibiotics and Control or from DEGs between MCE and Control were almost identical, including Th17 cell differentiation, butanoate metabolism, T-cell receptor signaling pathway, intestinal immune network for IgA production, antigen processing and presentation, and ABC transporters. Furthermore, an integrative analysis indicated that significant positive correlations (*p* < 0.05) were observed between *HEPHL1* and the featured biomarkers *Atopostipes*, *Syntrophococcus*, *Romboutsia*, and *Acinetobacter* in the MCE group. Conversely, several significant negative correlations (*p* < 0.05) were identified between *HEPHL1* and the featured biomarkers *Clostridium_XlVa*, *Phascolarctobacterium*, *Desulfovibrio*, *Cloacibacillus*, *Barnesiella*, *Succinatimonas*, *Alistipes*, *Oscillibacter*, *Ruminococcus2*, and *Megasphaera* in the Antibiotics group.

**Conclusion:**

Collectively, the analysis of microbiome–transcriptome data revealed that dietary supplementation with MCE produced significant alterations in multiple immune pathways, while having minimal impact on the microbial structure.

## Background

For decades, it has been a well-established practice by livestock producers to use subtherapeutic doses of antibiotics in animal feed to ward off “subclinical infections” that depress the activity of animal’s immune system and further to elevate the productive performance of modern animal husbandry (Van Boeckel et al., 2015). However, owing to the increasing concern about the emergence of antibiotic-resistant strains of bacteria, the European Commission decided to phase out and ultimately eliminate (1 January 2006) the use and marketing of antibiotics as growth promoters (Castanon, 2007). Subsequently, on 1 July 2020, legislation banning 11 antibiotics went into effect, ushering in a new age of non-antibiotic growth promoters in China (Wen et al., 2022). In the meantime, to overcome the considerable mortality and morbidity due to the ban of in-feed antibiotics, great efforts have been made by researchers to develop cheap, efficient, and environmentally alternatives to antibiotics, including Chinese veterinary drugs and natural plant extracts (Rahman et al., 2022). In addition to the characteristics of plant-derived products being natural, multi-functional, and low in toxicity, recent studies indicated that natural plant extracts also have positive effects in suppressing inflammation and improving immune function and animal performance (Di Sotto et al., 2020). Eco-friendly and residue-free, natural plant extracts are ideal substitutes for antibiotics in animal production.

*Macleaya cordata*, a perennial plant, has been considered as a traditional folk herbal medicine, which is mainly distributed in China, Europe, and North America (Vienna et al., 2007). Additionally, the phytobiotic macleaya cordata extract (MCE) has been widely used in feeding livestock and the effective chemical composition of MCE includes sanguinarine and chelerythrine, both belonging to a group of benzylisoquinoline alkaloids, which have detoxifying, analgesic, anti-inflammatory, antimicrobial, and antitumoral properties (Kosina et al., 2010; Lin et al., 2018). Previous studies using weaned piglets demonstrated that dietary supplementation of MCE reduced the diarrhea rate and enhanced the production performance through improvement of antioxidant capacity, immunity, and intestinal function (Wang et al., 2021). Studies with broiler chickens also showed increased growth performance that was associated with the modulation of intestinal microbiota by inhibiting the colonization of pathogenic bacteria including *E. coli* and *Salmonella* and increasing the abundance of beneficial bacteria including *Lactobacillus* and *Bifidobacterium* for MCE supplements (Huang et al., 2018). Given all this, the scientific issue of the effect of MCE on the production performance of animals by mediating intestinal bacterial communities and mucosal immune responses and their interaction mechanisms in the research fields of pigs and chickens has attracted extensive attention. However, the impact of MCE on the gut microbiota composition and immune reaction of ruminant animals is poorly understood. On the other hand, given the excessive drug residues in animal products and the emergence of drug-resistant bacteria caused by the long-term antibiotic use (Kapil, 2005), the antimicrobial properties of MCE do not have adverse effects on the intestinal microbiota. Therefore, new studies focusing on dietary MCE supplements to ruminants are needed to assess the impact of MCE on microbiota, immune responses, and their interplay.

Our previous parallel studies demonstrated that MCE significantly reduced the content of malondialdehyde and upregulated the activities of superoxide dismutase and glutathione peroxidase in the small intestine (Chen K. et al., 2020). Additionally, instead of the colon or jejunum, the quantitative real-time polymerase chain reaction (qRT-PCR) investigations of genes linked to immune-related pathways demonstrated that the primary site where the anti-inflammatory effects of MCE predominantly operated was the ileum (Yang et al., 2021). In addition, even though MCE does not contribute to antibiotic resistance gene dissemination, it could interfere with the structures and functions of the gut microbiome (Zhang et al., 2021). The intestinal epithelium, which is the primary interface between host and microbiota, is one of the major targets of antibiotic agents (Yu et al., 2017). In addition to being directly affected by antibiotics, the functions of intestinal mucosa can also be altered by the changing microbiota, especially those mucosa-attached bacteria (Song et al., 2018). Nevertheless, little is known about the impact of the host–microbiota interplay upon the supplementation of MCE on the interface of ileum of young goats. Hence, the objectives of this study were (1) to assess the impact of the supplementation of MCE on the structures and functions of mucosa-associated microbiota, (2) to explore the mucosal function response with the administration of MCE, and (3) to clarify the interaction between the host and microbiota.

## Materials and methods

### Animal management

A total of 30 female Xiangdong black goats (with an initial body weight 5.63 ± 0.30 kg and an average age 45 ± 2 days after birth) were selected and randomly allotted into one of the three diets: (1) a control diet was formulated to meet the recommendation of Lu et al. (1996) for young goats in China and its detailed information was described by our previous study (Yang et al., 2021); (2) a control diet supplemented with 21 mg/kg/day vancomycin and 42 mg/kg/day neomycin; and (3) a control diet supplemented with 0.3 g/day MCE (Sangrovit®, MCE 3.75% w/w premix, Changsha, China). The supplementing rates of additive used in this study were suggested by previous studies (Chen K. et al., 2020; Yang et al., 2021). Goats were fed in total mixed diets twice daily at 8:00 and 17:00 h in amounts to ensure less than 10% orts and housed in individual pens (width × length × height, 1.00 m × 1.20 m × 0.75 m) throughout the experiment. The amount of the antibiotic additive or MCE additive was top-dressed every day to make sure that the goats could consume the entire additive. During the pre-feeding period, which spanned 2 weeks, and the subsequent formal feeding trial that lasted for 5 weeks, all goats were provided with unrestricted access to water.

### Sample collection

Anesthesia was carried out by intravenous injection of sodium pentobarbital (50 mg kg^−1^ BW), and then the goats were exsanguinated 4 h after the morning feeding; the middle part of the ileum was collected and immediately rinsed with ice-cold saline (0.9% sodium chloride solution) (Wu et al., 2022). Then, the mucosa was quickly shaved off using glass slides within 15 min after the goats underwent exsanguination. Afterwards, these mucosae were rapidly wrapped with sterilized tinfoil, frozen in liquid nitrogen, and stored at −80°C in a refrigerator for follow-up RNA-Seq and amplicon sequencing.

### DNA extraction, 16S rDNA amplicon sequencing, and bioinformatics analysis

Approximately 0.20 g of ileal mucosa was used to extract total metagenomic DNA from all the samples using QIAamp DNA Stool Mini Kits (Qiagen GmbH, Hilden, Germany) with some modifications according to our previous study (Wu et al., 2022). Briefly, a bead-beating bacterial cell wall disruption procedure was added firstly followed by lysis at 85°C for 10 min. The DNA concentration was measured using a Nanodrop ND-2000 spectrophotometer (Thermo Scientific, Wilmington, MA,USA), and DNA integrity was verified using a BioAnalyzer 2100 (Agilent, Palo Alto, CA, USA). To study variations in microbial community structure and composition, the PCR was conducted to amplify the V3 and V4 regions of the bacterial 16S rRNA gene using universal primer 341F (5′-CCTAYGGGRBGCASCAG-3′) and 806R (5′-GACTACNNGGGTATCTAAT-3′). The PCR conditions were detailed in our previous study (Jiao et al., 2016). Then, the products were quantified and pooled in equal molar amount. The amplification products were purified with QIAquick Gel extraction Kit (Qiagen). Afterward, the sequencing was operated on an Illumina HiSeq 2500 platform.

The barcodes and sequencing primers were removed before data processing. The sequences with a similarity level of more than 99% was clustered into amplicon sequence variants (ASVs) using the method of unoise3 by VSEARCH v.2.7.1 (Rognes et al., 2016) based on the reference database of SILVA v.132 (Quast et al., 2012). Afterwards, the representative sequences were submitted to the RDP classifier v.16 to obtain the taxonomy assignment with a 0.80 confidence threshold (Wang et al., 2007). Analyses of alpha and beta diversities were performed in R using packages microeco v0.8.0 (Liu C. et al., 2021). Principal coordinate analysis (PCoA) with the Bray–Curtis dissimilarities and Jaccard index was used to explore microbial community structure. Permutational multivariate ANOVA (PERMANOVA) was performed for beta diversity analysis. Furthermore, to obtain the distinct microbiota biomarkers, random forest classification was performed with the groups at the genus level using R “random Forest” package v4.7-1.1 (Liaw and Wiener, 2002). The number of trees and features sampled for each split of the tree both applied the default parameters. Moreover, MeanDecreaseGini is selected as the indicator value in the analysis. Furthermore, BugBase analysis was performed using the “run.bugbase.r” pipeline to predict the proportions of microorganisms with specific characteristics such as mobile elements, biofilm-forming capability, oxidative stress tolerance, and potential pathogenicity (Ward et al., 2017).

### RNA extraction, transcriptome sequencing, and bioinformatics analysis

Total RNA was extracted from the ileal mucosa using the TaKaRa MiniBEST Universal RNA Extraction Kit (TaKaRa, Dalian, China; Code No. 9767) according to the manufacturer’s instructions. Then, RNA quality was determined using the 2100 Bioanalyzer (Agilent Technologies, Santa Clara, USA). High-quality RNA samples (RIN ≥ 7) were used to construct sequencing library following the TruSeqTM RNA Sample Preparation Kit from Illumina (San Diego, CA), using 1 μg of total RNA. Shortly, messenger RNA was isolated with polyA selection by oligo (dT) beads and fragmented using fragmentation buffer. The cDNA synthesis, end repair, A-base addition, and ligation of the Illumina-indexed adaptors were performed according to Illumina’s protocol. Libraries were size selected for cDNA target fragments of 200–300 bp on 2% low range ultra-agarose and then PCR amplified using Phusion DNA polymerase (NEB) for 15 PCR cycles. After being quantified by TBS380, paired-end libraries were sequenced by Illumina NovaSeq 6000 sequencing.

The raw paired-end reads were trimmed and quality controlled by Trimmomatic (Version 0.36) with parameters (SLIDINGWINDOW:4:15 MINLEN:75) (Bolger et al., 2014). Then, clean reads were separately aligned to goat genome reference (ARS1) (https://ftp.ncbi.nlm.nih.gov/genomes/all/GCF/001/704/415/GCF_001704415.1_ARS1/GCF_001704415.1_ARS1_genomic.fna.gz) using HISAT2 software with default parameters (Kim et al., 2019). The quality assessment of these data was taken by Qualimap 2 (Okonechnikov et al., 2016). Use HTSeq 2.0 to count each gene read (Anders et al., 2015). Read counts were normalized and transformed using regularized log (rlog) transformation using the DESeq2 R package (version 1.30.1) (Love et al., 2014). PCoA of Bray–Curtis distance based on rlog data was calculated applying the ape package (version 5.5) and visualized in R (Version 4.0.4) (Paradis and Schliep, 2019). The identification of differentially expressed genes (DEGs) was determined by DESeq2 using FDR < 0.05 and |log2FC| > 1.2 as the cutoff. Then, the DEGs were used to perform pathway enrichment analysis and visualization using the R package clusterProfiler (Version 4.0) (Wu et al., 2021).

### Integrated analysis of interactions between changes in differential microbiome and host genes

Transcriptomic DEGs and biomarker microbiota were used for integrated analysis. Firstly, we selected the subset of 135 shared DEGs between Antibiotics vs. Control and Macleaya vs. Control, for subsequent KEGG pathway enrichment. Then, we obtained a representative set of both up- and downregulated genes from pathways related to the immune system, metabolism, digestive system, and signal transduction, leaving 36 genes for downstream analysis. Secondly, as described above, differentially abundant microbial taxa were selected using the random forest method. Representative gene–taxa correlations were visualized using corrplots in R (Wei et al., 2017), where the strength of the correlation is indicated by the color and size of the visualization element (square) and the significance of the correlation is indicated by an asterisk.

## Results

### The microbial features

**Figure 1:**
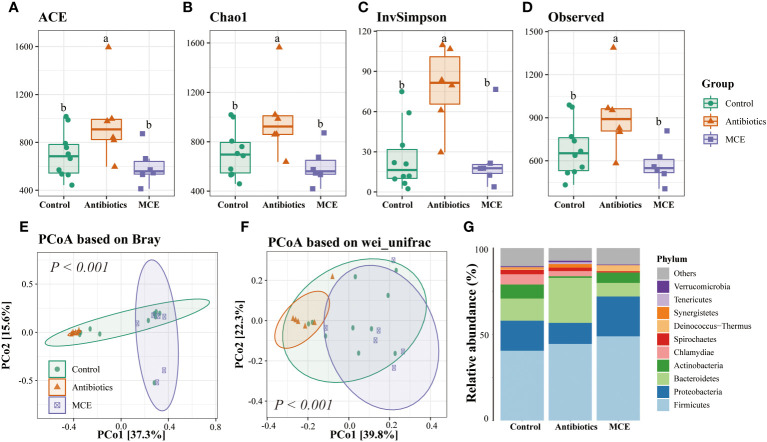
The alpha diversity of Control (blue), Antibiotics (red), and Macleaya (purple) groups was depicted through box plots based on the ACE **(A)**, Chao1 **(B)**, InvSimpson **(C)**, and Observed **(D)** indices. Principal coordinate analysis (PCoA) derived from **(E)** Bray and **(F)** weighted UniFrac distances among samples of the three groups. The relative abundance of microbiota at the phylum level **(G)**. The same letters mean no significant difference (*p* > 0.05), while different letters mean significant difference (*p* < 0.05).

**Figure 2:**
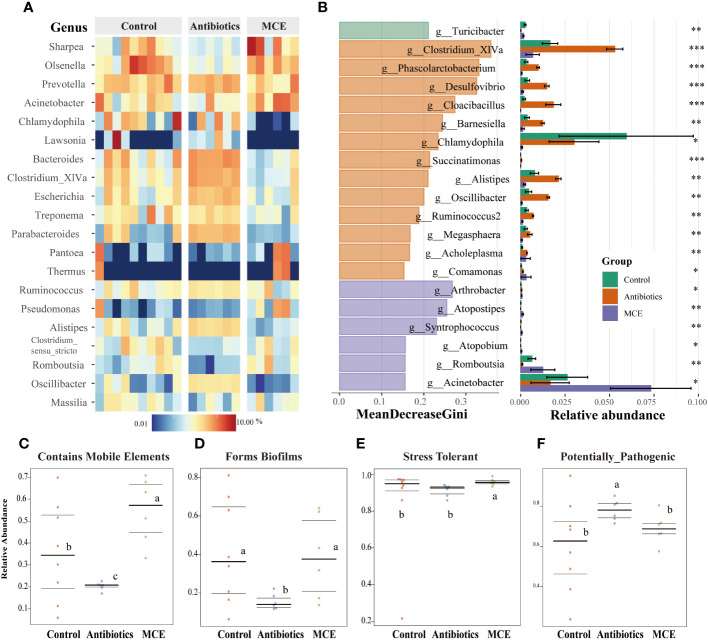
Heatmap plot **(A)** of the relative abundances of predominant bacterial taxa at the genus level (top 20) in each sample among groups. Bacterial biomarkers selected based on the rankings (top 20) of Mean Decrease Gini generated by Random Forest and their relative abundance (*, 0.05< *p* < 0.1, **, 0.01< *p* < 0.05, ***, *p* < 0.01 ) **(B)**. BugBase phenotypes prediction: **(C)** Contains Mobile Elements, **(D)** Forms Biofilms, **(E)** Stress Tolerant, and **(F)** Potentially Pathogenic. The phenotype relative abundances were compared using pairwise Mann–Whitney *U* tests with false discovery rate correction. The same letters mean no significant difference (*p* > 0.05), while different letters mean significant difference (*p* < 0.05).

Microbial alpha diversity analysis revealed that the indexes including ACE, Chao1, InvSimpson, and Observed were not significantly different between Control and MCE, but significantly greater in the Antibiotics group than in the other two groups (**Figures 1A–D**). We next assessed the dissimilarities among groups using the Bray–Curtis dissimilarity and weighted UniFrac distance metrics to evaluate the overall differences in beta diversity. The PCoA showed that samples of the Antibiotics group clustered separately from both Control and MCE (**Figures 1E, F**). As shown in **Figure 1G**, the *Firmicutes*, *Bacteroidetes*, and *Proteobacteria* were the top three most abundant phyla, with statistically differential distribution among groups. Specifically, the relative abundance of *Bacteroidetes* was dramatically greater (*p* < 0.05), while that of *Actinobacteria* was lower (*p* < 0.05) in the Antibiotics when compared to other groups. Moreover, the Antibiotic-treated group had a negative impact on the abundance of *Sharpea*, which is known as a well-established lactic acid producer, and was conspicuously lower (*p* < 0.05) than the Control and MCE groups (**Figure 2A**). In contrast, the Antibiotics group exhibited significant enrichment of putative pathogens, including taxa assigned to *Proteobacteria*, *Bacteroidetes*, *Treponema*, and *Escherichia*.

To further screen for differential microorganisms, we employed random forest analysis to assess the relative importance of each feature by the value of MeanDecreaseGini. As shown in **Figure 2B**, 13 microbial members were filtered out with the administration of antibiotics. *Clostridium XIVa*, which has been shown to aid in the expansion of anti-inflammatory Treg cells via butyrate production, was the most important and abundant in the Antibiotics group. The abundance of other featured biomarkers, such as *Phascolarctobacterim*, *Desulfovibrio, Cloacibacillus, Barnesiella*, and *Oscillibacter*, was upregulated twofold with Antibiotics treatment compared to the Control. In contrast, the abundance of four genera (*Arthrobacter*, *Atopostip*es, *Syntrophococcus*, and *Atopobium*) was less than 1% and selected as featured biomarkers in the MCE group. Notably, community functional mapping using the Bugbase indicated that the Antibiotics group significantly downregulated the mobile elements, which propagate repetitive elements that can move through the genome (**Figure 2C**). Furthermore, Antibiotics treatment decreased the ability of the microorganism to form biofilms and increased the abundance of potentially pathogenic microbes (**Figures 2D, F**). In contrast, MCE administration memorably enhanced the ability of tolerance to oxidative stress (**Figure 2E**). Therefore, although MCE treatment slightly altered the community structure, the functional changes were deemed to be beneficial.

### Differentially expressed genes in the ileal mucosa across groups

**Figure 3:**
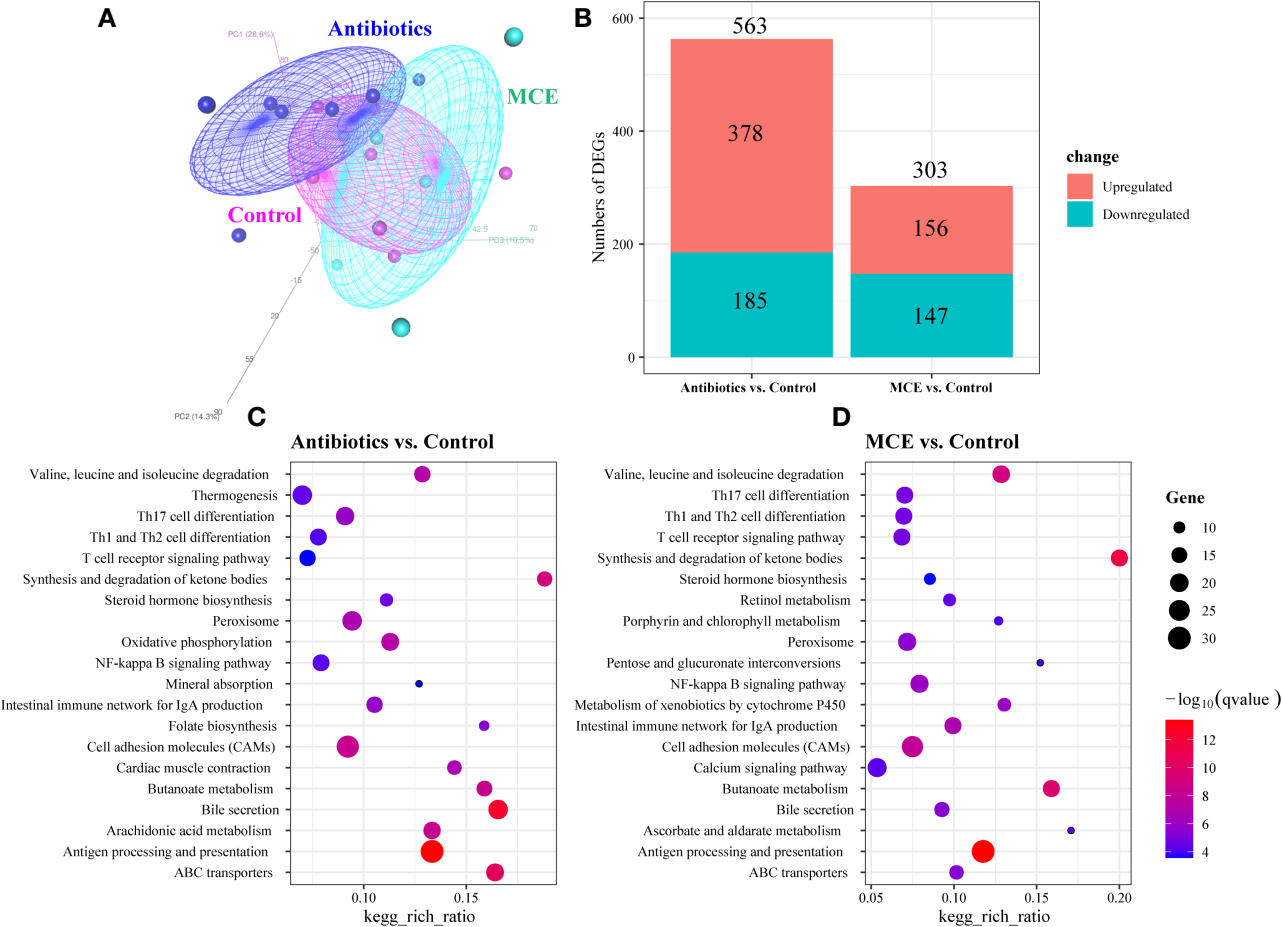
Principal coordinate analysis (PCoA) of ileal mucosa transcriptome based on Bray–Curtis distance **(A)**. Bar plots showing the numbers of upregulated DEGs (colored in red) and downregulated DEGs (colored in blue) between groups **(B)**. KEGG pathway analysis of the DEGs identified in comparison to Antibiotics treatment with control **(C)**. KEGG pathway analysis of the DEGs identified in comparison to Macleaya treatment with control **(D)**. The *X*‐axis represents the ratio of DEGs corresponding to the KEGG pathway. The *Y*‐axis represents the KEGG pathway. The size of the dot represents counts, and the color of the dot represents adjusted *p*-value.

**Figure 4:**
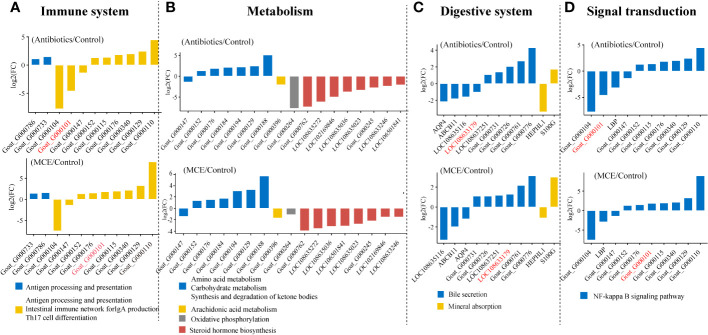
Gene expressions in main KEGG pathways of shared differentially expressed genes in both “Antibiotics vs. Control” and “MCE vs. Control” **(A–D)**.

The PCoA plots of total gene expression in ileal mucosa samples showed an overlap between the expression profile of most samples from the Control and MCE group, and distinct separation between Antibiotics and the other two groups (**Figure 3A**). The Antibiotics group exhibited 563 DEGs (378 upregulated and 185 downregulated; *q*-value <0.05) in comparison to the Control group (**Figure 3B**). The MCE group displayed 303 DEGs (156 upregulated and 147 downregulated) relative to the Control group. The enriched KEGG pathways from the significant DEGs between the Antibiotics and Control groups, as well as between the MCE and Control groups, were almost identical, including Th17 cell differentiation, butanoate metabolism, T-cell receptor signaling pathway, intestinal immune network for IgA production, antigen processing and presentation, and ABC transporters (**Figures 3C, D**). Meanwhile, the shared DEGs between “Antibiotics vs. Control” and “MCE vs. Control” in those pathways also exhibited similar trends (**Figures 4A–D**). To be more precise, out of those common DEGs associated with immune response, metabolism, digestive system, and signal transduction, the trends in their alterations were analogous, except for *Goat_G000101* and *LOC108633179*, indicating that there is minimal distinction in transcriptional regulation.

### Interactions between bacterial biomarkers and shared DEGs

**Figure 5:**
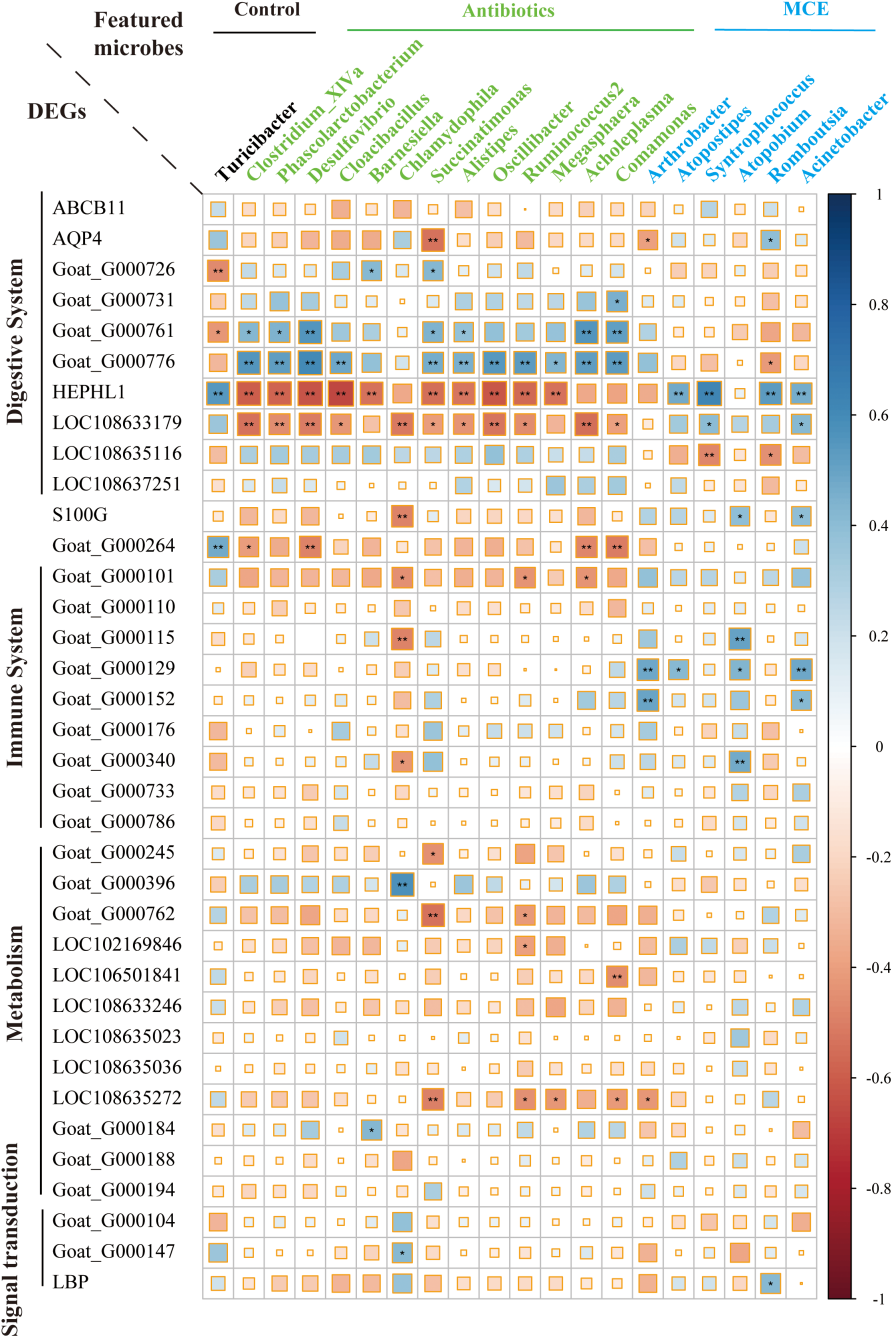
Correlation plot depicting gene–microbe correlations. Color and size of the squares indicate the magnitude of the correlation, and asterisks indicate significance of correlation (** indicates *q*-value < 0.05 and * indicates *q*-value < 0.1).

To elucidate the host–microbiota interplay in the ileal mucosa, we examined the correlations between enriched DEGs in response to both Antibiotics and MCE treatments, and the featured microbes identified by random forest analysis. Through Spearman correlations, we identified that digestive system-related DEGs such as *Goat_G000761*, *Goat_G000776*, *HEPHL1*, and *LOC108633179* exhibited a stronger correlation with the featured microbes than other genes (**Figure 5**). Interesting findings revealed opposite microbe–gene correlations in response to Antibiotics or MCE treatments. Significant positive correlations (*p* < 0.05) were observed between *HEPHL1* and the featured biomarkers *Atopostipes* (*r* = 0.48), *Syntrophococcus* (*r* = 0.62), *Romboutsia* (*r* = 0.53), and *Acinetobacter* (*r* = 0.47) in the MCE group. Conversely, several significant negative correlations (*p* < 0.05) were identified between *HEPHL1* and the featured biomarkers *Clostridium_XlVa* (*r* = −0.59), *Phascolarctobacterium* (*r* = −0.58), *Desulfovibrio* (*r* = −0.62), *Cloacibacillus* (*r* = −0.67), *Barnesiella* (*r* = −0.54), *Succinatimonas* (*r* = −0.54), *Alistipes* (*r* = −0.53), *Oscillibacter* (*r* = 0.61), *Ruminococcus2* (*r* = −0.57), and *Megasphaera* (*r* = −0.54) in the Antibiotics group.

## Discussion

The abuse of antibiotics and their association with the antibiotic-resistant strains of bacteria are receiving global attention (Zhang et al., 2021). MCE, as an antibiotic alternative, is being developed in feed livestock and is widely used in monogastric animals (Di Sotto et al., 2020; Liu et al., 2020; Lei et al., 2021). However, the poor understanding of the molecular mechanisms induced by MCE impedes its large-scale use in ruminants. Furthermore, revealing the microbiota–host interplay in the context of plant extracts replacing antibiotics is crucial for the development of knowledge-based strategies to enhance animal productivity and host health (Liu K. et al., 2021). Herein, we firstly observed a slight shift in the ileal microbiome and identified taxa with the administration of MCE compared to antibiotics using the amplicon approach. We also observed some common altered genes and singling pathways induced by both MCE and antibiotics with the means of transcriptome. Finally, we further found relevant correlations between these microbes and genes that may illuminate the mechanisms underlying the growth and development of ruminants.

To date, numerous studies have shown that microbes possess great potential contributions to animal husbandry (McCann et al., 2017). The addition of antibiotics alters the mucosal microbiome of ruminants both taxonomically and functionally (Patil et al., 2021). A greater microbial taxonomic diversity in goats fed antibiotics was observed in this study, most likely because using subtherapeutic doses of antibiotics as growth promoters led to a more even taxonomic profile (Patil et al., 2021; Wang et al., 2021). This was also confirmed by the dissimilar distance analysis, showing that the homogeneity within the Antibiotics group was notably reduced, whereas administering MCE slightly altered the taxonomic diversity and within-group distance, suggesting that MCE might not contribute to the emergency of antibiotic resistance (Zhang et al., 2021). Knowledge from random forest analysis identified 13 and 6 bacterial genera as marker microorganisms in Antibiotic and MCE groups, respectively. Furthermore, the abundance of only two marker microorganisms (*Romboutsia* and *Acinetobacter*) exceeded 1%, indicating that MCE slightly altered the mucosa-attached microbiota in the ileum. *Clostridium_XIVa* has been proposed as butyrate producers (Van den Abbeele et al., 2013), and its pronounced upregulation by administering a low dose of antibiotics might enhance butyrate bioavailability and thereafter promote growth (Van den Abbeele et al., 2013). On the other hand, the abundance of *Phascolarctobacterium*, which mainly produces propionic acid by fermentation of succinate (Watanabe et al., 2012), was also increased by administering a low dose of antibiotics. It has been suggested that stimulating propionate production by increasing the abundance of propionic acid bacteria is another novel way to enhance the ruminal feed degradation and reduce the methane production (Chen J. et al., 2020). Insights from BugBase phenotypic prediction (Ward et al., 2017) indicated that the MCE group had lower potentially pathogenic microflora abundance and stronger oxidative stress tolerability, while antibiotics strikingly decreased the quantity of mobile elements and the capability of forming biofilms. Although the administration of subtherapeutic antibiotics could improve the abundance of propionate- and butyrate-producing bacteria, as well as increase the ability of being potentially pathogenic, MCE treatment slightly altered the composition of microbiota and improved the oxidative stress tolerability (Robinson et al., 2019).

In addition to microbiota, the host genes have also been implicated in driving the physiological processes induced by growth-promoting antibiotics (Wang et al., 2021). It is worthy to note that although the number of DEGs affected varied with the administration of antibiotics or MCE, the signaling pathways enriched by those DEGs were largely consistent, suggesting that MCE can effectively mimic some of the changes induced by low-dose antibiotics. While pathogen inhibition has been found to be a long-established contributor to the efficacy of antibiotic growth promoters, other mechanisms have also been proposed, including anti-inflammatory and immunostimulatory effects at the gut level (Ward et al., 2019). Our findings make a strong case for this proposal, as several pathways associated with the immune system were enriched with the administration of antibiotics or MCE, including antigen processing and presentation, intestinal immune network for IgA production, and Th17 cell differentiation (Yang et al., 2021). Low et al. (2021) suggested that the primary function of antibiotics was to improve gut barrier function by tamping down wall inflammation and improving nutrient absorption. In addition, Cui et al. (2023) also figured out that various mechanisms have been proposed as possible explanations on how subtherapeutic levels of antibiotics improved growth in farm animals, comprising the digestion and metabolism systems of nutrients and the signal pathways involved (Low et al., 2021). In the current study, pathways that were significantly altered upon administration of both antibiotics and MCE were also primarily associated with the metabolic and digestive systems and the signal transduction. Similar to other studies that suggested that antibiotics treatment increased the relative concentrations of metabolites involved in amino acid metabolism (Mu et al., 2017), the genes involved in amino acid metabolism were upregulated by antibiotics or MCE. The biosynthesis pathway of steroid hormones, which plays key roles in anti-inflammation (Vasconcelos et al., 2016), was weakened to reduce unnecessary functional redundancy. In addition, the expression changes of some genes in the digestive system and signal transduction were consistent between the two groups. Therefore, at the level of gene transcription, the regulatory mechanism between the low-dose antibiotics and the MCE may be somewhat identical.

Integrating mucosal microbiome and host gene expression profiles, we observed several correlations between differentially expressed epithelial genes and mucosal bacteria. *Chlamydophila*, a controversial bacterial genus belonging to the family *Chlamydiaceae* that has the potential to cause tract infection (Gieffers et al., 2001), was positively associated with the immune system. Interestingly, we found that *Chlamydiaceae* was in higher abundance in both treatments compared to the control, hinting that this microbe may be the key hallmarked bacteria to activate the immune system by low-dose antibiotics or MCE. Moreover, these immunological processes may also be promoted by the higher expression of *S100G* in Antibiotics, which correlates with calcium transport activity of the reaction cascade (Wang et al., 2004). Hephaestin-like 1 (*HEPHL1*) is a new member of the multicopper oxidase family, which plays a critical role in maintaining iron homeostasis and predominantly expressed in the basolateral membrane of absorptive intestinal cells (Sharma et al., 2019). Furthermore, our findings show that many bacteria including *Clostridium_XIVa*, *Ruminococcus2*, and *Desulfovibrio* have a strong negative correlation with *HEPHL1* in Antibiotics, while *Atropostipes*, *Syntrophococcus*, *Romboutsia*, and *Acinetobacter* have a positive correlation with *HEPHL1* in MCE, which implied that multiple bacteria collaborate to regulate this key target. Surprisingly, a novel gene (*Goat_G000776*) was excavated and probably plays an opposite role to *HEPHL1* in light of the significantly negative correlation with those bacteria, and the specific function of this gene warrants further investigation.

## Conclusions

Antibiotics dramatically altered the microbial community structure, as well as influenced host genes implicated in immune response, nutrition metabolism, and signal transduction; the microbe–gene interplay worked to achieve the growth promotion. Nevertheless, the degree of influence of MCE on the ileal microbiome is relatively small, indicating that it mainly affected growth and development by regulating the gene expression of the host.

## Data availability statement

The datasets presented in this study can be found in online repositories. The names of the repository/repositories and accession number(s) can be found in the article/supplementary material.

## Ethics statement

The animal studies were approved by the Animal Care Committee of Institute of Subtropical Agriculture. The studies were conducted in accordance with the local legislation and institutional requirements. Written informed consent was obtained from the owners for the participation of their animals in this study.

## Author contributions

JW: Data curation, Formal Analysis, Methodology, Visualization, Writing – original draft, Writing – review & editing. XZ: Methodology, Writing – review & editing. CZ: Conceptualization, Investigation, Writing – review & editing. JJ: Methodology, Validation, Writing – review & editing. ZT: Conceptualization, Data curation, Software, Writing – review & editing.
